# A burns and COVID-19 shared stress responding gene network deciphers CD1C-CD141- DCs as the key cellular components in septic prognosis

**DOI:** 10.1038/s41420-023-01518-7

**Published:** 2023-07-24

**Authors:** Qiao Liang, Lei Wang, Jing Xu, Anqi Lin, Yongzheng Wu, Qing Tao, Bin Zhang, Haiyan Min, Shiyu Song, Qian Gao

**Affiliations:** 1 Center for Translational Medicine and Jiangsu Key Laboratory of Molecular Medicine, Medical School of Nanjing University, Nanjing, 210093 Jiangsu Province China; 2 Department of Clinical Laboratory, Jiangsu Provincial Hospital of Integrated Chinese and Western Medicine, Jiangsu Province Academy of Traditional Chinese Medicine, Nanjing, 210028 China; 3 Central Laboratory, Nanjing Chest Hospital, Nanjing Medical University, Nanjing, 210028 China; 4 Central Laboratory, The Second Affiliated Hospital of Nanjing University of Chinese Medicine, Nanjing, 210028 China

**Keywords:** Viral infection, Sepsis, Cell death and immune response

## Abstract

Differential body responses to various stresses, infectious or noninfectious, govern clinical outcomes ranging from asymptoma to death. However, the common molecular and cellular nature of the stress responsome across different stimuli is not described. In this study, we compared the expression behaviors between burns and COVID-19 infection by choosing the transcriptome of peripheral blood from related patients as the analytic target since the blood cells reflect the systemic landscape of immune status. To this end, we identified an immune co-stimulator (CD86)-centered network, named stress-response core (SRC), which was robustly co-expressed in burns and COVID-19. The enhancement of SRC genes (SRCs) expression indicated favorable prognosis and less severity in both conditions. An independent whole blood single-cell RNA sequencing of COVID-19 patients demonstrated that the monocyte-dendritic cell (Mono-DC) wing was the major cellular source of SRC, among which the higher expression of the SRCs in the monocyte was associated with the asymptomatic COVID-19 patients, while the quantity-restricted and function-defected CD1C-CD141-DCs were recognized as the key signature which linked to bad consequences. Specifically, the proportion of the CD1C-CD141-DCs and their SRCs expression were step-wise reduced along with worse clinic conditions while the subcluster of CD1C-CD141-DCs from the critical COVID-19 patients was characterized of IFN signaling quiescence, high mitochondrial metabolism and immune-communication inactivation. Thus, our study identified an expression-synchronized and function-focused gene network in Mono-DC population whose expression status was prognosis-related and might serve as a new target of diagnosis and therapy.

## Introduction

Stressors can elicit complex and dynamic responses in the host, depending on the severity, duration and nature of the stimulus, leading to a range of outcomes from asymptomatic to fatal [1]. However, the underlying mechanisms governing stress responses that determine clinical outcomes remain unclear. The cooccurrence of hyper-inflammation and hypo-immunity, e.g. T cell exhaustion, represents the most severe form of dysregulation, often leading to the worst prognosis with multiple organ damage and failure (MOF), systemic inflammatory response syndrome (SIRS), or sepsis. The disrupted immune homeostasis, involving alterations in specific cell types, their quantity and molecular dysfunction, could be the critical and common step underlying the progression of various stresses [2–5].

Burns are mainly characterized by serious destruction of the skin and related deeper tissues, and are often complicated by MOF, SIRS and sepsis, which leads to nearly 180,000 deaths each year [6, 7]. The immune dysregulation is a common feature of severe burns which not only occurs in affected tissues but also system [8, 9]. The burn victims with T cell immunosuppression demonstrate increasing susceptibility to septic complications, which is characterized by a combination of T cell depletion, reduction of proliferation, dysfunction and anergy [10–14]. Although numbers of factors have been reported to be involved in T cell immunosuppression [15–19], the precise molecular mechanism by high throughput sequencing remains not fully investigated.

COVID-19 pandemic has led to more than 614 million cases and 6.5 million deaths worldwide (https://coronavirus.jhu.edu/map.html). The heterogeneous outcomes of it suggested the existence of differential stress responses of individuals [20–22]. A compromised immune response leads to worse outcomes, characterized by sustained innate immune activation and insufficient adaptive immune response, indicating the key importance of the transition between innate and adaptive immune response in stress prognosis. In fact, in the severe COVID-19 patients and aging individuals, reduced numbers and altered activation of dendritic cells (DCs) were observed, with compromised CD80 and CD86 co-stimulatory molecules, which are necessary for T cell proliferation and activation [23–27]. The recovery of DCs number and function by targeting these molecules is a promising mean to reduce mortality in COVID-19 related sepsis [27], while CD4+ and CD8 + T cells are responsible for SARS-CoV-2 clearance via MHC-II and MHC-I, respectively, and B cell-produced antibodies are enhanced by CD4 + T cells, further promoting CD8 + T-cell-mediated cytotoxicity [28–31].

Herein, we hypothesized that there might be a shared molecular pattern with designated cellular dysfunctions between burns and COVID-19 infection, which is responsible for worse clinic outcomes. We identified a co-expressed gene signature, referred to stress-response core genes (SRCs), which was robust in gene membership and indicated less disease severity in burn injury and COVID-19. The SRCs were mainly expressed in monocytes/DCs upon COVID-19 single-cell RNA sequencing (scRNA-seq) results, among which the CD1C-CD141-DCs exhibited a relationship with COVID-19 severity and outcome. The subcluster of these DCs from critical COVID-19 patients demonstrated an “uninfected” molecular profile of compromised IFN signaling and was quiescent in intercellular interaction with other immune components upon silico analysis.

## Results

### Identify a clinically relevant gene co-expression network in burns datasets

**Fig. 1:**
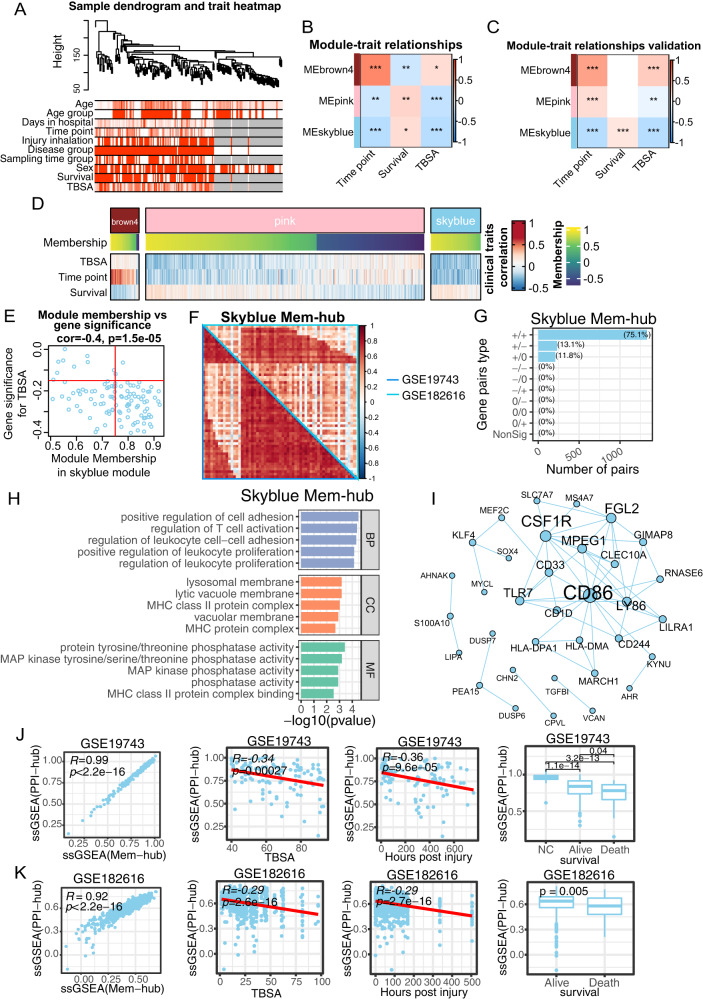
CD86-centered gene network was the robust and prognostic core signature of burns systematic alternation. **A** Clustering dendrogram of samples in burns dataset (GSE19743) based on expression pattern. The clinical information was represented by red color whose intensity was proportional to older age, longer hospital-stay days, later time point of sample-collection, injury inhalation, burns condition, male, survival as well as larger TBSA. Representative heatmap of vital module eigengenes and clinical trait correlation in GSE19743 (**B**) and GSE182616 (**C**). **D** Heatmap of genes membership and clinical-traits correlation in the vital modules. **E** Skyblue module genes membership and correlation with TBSA. The red line represented the threshold of mem-hub pick-up which was 0.75 and -0.15 for membership and gene significance for TBSA, representatively. **F** Pearson correlation heatmap of skyblue mem-hub genes of GSE19743 and GSE182616 datasets. The left-bottom and right-top part of heatmap is based on GSE19743 and GSE182616, respectively. **G** Barplot of DGCA based on skyblue mem-hub genes between GSE19743 and GSE182616. Each gene pair is classed as positive (+), negative (−) and non-significant (0) and grouped by combination of GSE19743 and GSE182616 datasets. **H** PPI Network of skyblue mem-genes. The size of node and gene label represent the degree of specific node. (**I**) Barplot of top skyblue mem-hub genes GO enrichment items ordered by *p* value. SsGSEA scores of skyblue PPI-hub genes and mem-hub and correlation (left), as well as correlated with TBSA (middle-left), time point (middle-right) and survival (right) in GSE19743 (**J**) and GSE182616 (**K**). Pearson and Spearman correlation was applied for PPI-hub with mem-hub and clinical traits, respectively. *P < 0.05, **P < 0.01, ***P < 0.001.

We applied weighted gene co-expression network analysis (WGCNA) to identify clinical-related gene co-expression modules in burns peripheral blood cell transcriptome dataset GSE19743 (see the workflow in Supplementary Fig. S1). A total of 114 burns patients and 63 healthy controls were separated into two clusters under the dendrogram clustering (Fig. 1A), indicating that burns extremely shifted the gene expression. With a scale-free network, 52 modules were generated according to the hierarchical clustering tree and automatic module detection (Supplementary Fig. S2A). The modules that were correlated with all of total body surface area (TBSA) injured percentage, survival status and hours post burns were selected, which left three modules namely brown4, pink and skyblue (Fig. 1B, D). The eigengenes of pink and skyblue modules were both negatively and positively correlated to TBSA and survival status, respectively, demonstrating that these two modules stood for good prognosis, while brown4 showed the opposite (Fig. 1B, D). Remarkably, the skyblue module was uniquely associated with stress-related characteristics, displaying no correlation with other clinical variables such as age or gender (Supplementary Fig. S2A).

We cross-validated the above findings in another burns transcriptome. The gene pair correlation in brown4 and skyblue modules between the two datasets illustrated a more conserved pattern than pink module, where 65% and 65.1% compared to 40.5% (Supplementary Fig. S2B, C). As for module-trait correlation, only skyblue module stayed consistent relationship with all of TBSA, survival status and time post injury (Fig. 1C). We thus selected the skyblue module for advanced analysis because of its robustness in both module membership and clinical relevance.

We then applied membership-traits filtering and protein-protein interaction (PPI) filtering to identify the hub genes in the skyblue module. We first picked up 60 hub genes in skyblue as mem-hub with threshold of membership >0.75 and gene significance for TBSA < −0.15 (Fig. 1E). As expected, the mem-hub genes were more robust between two burn datasets compared to those of the skyblue module genes (Fig. 1F, G). The Gene Ontology (GO) enrichment analysis revealed that the mem-hub was highly involved in both innate and adaptive immune processes, including T cell and leukocyte activation and proliferation and MHC complex formation (Fig. 1H). And, the mem-hub further formatted a compact PPI network with a few scattered connections (Fig. 1I).

We eventually extracted 24 genes in the major PPI network and referred them as PPI-hub. The consistent gene pair in PPI-hub in the two datasets increased from 75.1% to 85.5% compared to those between mem-hubs (Supplementary Fig. S2D). The ssGSEA scores of mem-hub and PPI-hub were highly correlated between the two burns datasets (Pearson correlation index > 0.9) (Fig. 1J, K), suggesting a further condensation of function-related genes in the PPI-hub. Clinically, the PPI-hub score was negatively correlated with TBSA and time post injury, suppressed in burns patients and further aggravated in dead ones (Fig. 1J, K).

### Network genes identified in burns were conserved in COVID-19

**Fig. 2:**
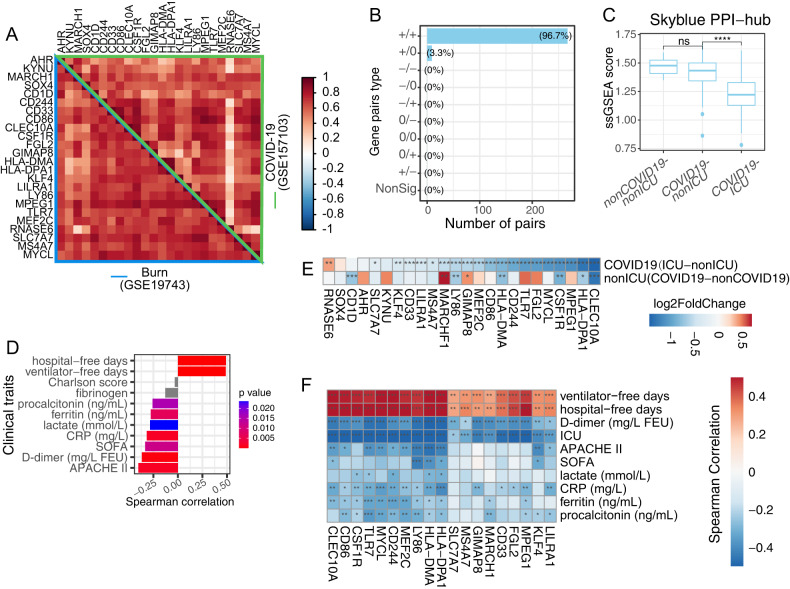
Burns’ core gene net was conserved in both membership and clinical-trait correlation in COVID-19. **A** Pearson correlation heatmap of skyblue PPI-hub genes in burns (GSE19743) and COVID-19 (GSE157103) datasets. The left-bottom and right-top represent burns and COVID-19 datasets and marked by blue and green color, respectively. **B** Barplot of skyblue PPI-hub gene pairs differential correlation on burns and COVID-19 datasets based on DGCA analysis. Each gene pair is classed as positive (+), negative (−) and non-significant (0) and grouped by combination of GSE19743 and GSE157103 datasets. **C** Boxplot of ssGSEA sore of skyblue PPI-hub genes across disease conditions in COVID-19 GSE157103 datasets. **D** Barplot of Spearman correlation index of skyblue PPI-hub ssGSEA score with COVID-19 patients clinical traits. The bar color represents the Spearman correlation *p* values and *p* ≥ 0.05 was colored by grey. **E** DEG log2FoldChange heatmap of skyblue PPI-hub genes in GSE157103 dataset. The color indicated the log2FoldChange in each disease status comparison. **F** Spearman correlation coefficient heatmap of SRC genes in GSE157103 dataset. *P < 0.05, **P < 0.01, ***P < 0.001.

Next, we examined the performance of the burn-origin PPI-hub in COVID-19 transcriptome to test the presence of a common signature responding to different stresses. Impressively, the PPI-hub demonstrated a highly synchronous pattern with 96.7% gene pairs maintained the positive correlation with burns data (GSE19743) (Fig. 2A, B), emphasizing the PPI-hub as the shared transcription signature of burns and COVID-19. Notably, PPI-hub score was specifically and dramatically restrained in severe COVID-19 patients rather than mild ones, and negatively correlated with disease severity (Fig. 2C, D). Specifically, the higher PPI-hub score implied the shorter hospitalizations and less ventilator, while the COVID-19-severity-related information, including acute physiologic assessment and chronic health evaluation (APACHE II) score, sequential organ failure assessment (SOFA) score, and laboratory measurements of C-reactive protein, D-dimer, ferritin, lactate and procalcitonin, claimed a negative correlation with ssGSEA score of PPI-hub (Fig. 2D).

We further simplified PPI-hub by drawing the 19 lower expressed genes in COVID-19 ICU vs mild COVID-19 (Fig. 2E), referring them as the stress response core genes (SRCs). Importantly, the individual genes of SRC were also broadly correlated with clinical traits such that the higher expression of which implied the lesser severity of the disease (Fig. 2F). These findings supported the notion that SRC is a shared gene signature in the burns and COVID-19. The higher expression of which predicts a good prognosis.

### COVID-19 scRNA-seq analysis revealed SRCs monocyte-DC specific

**Fig. 3:**
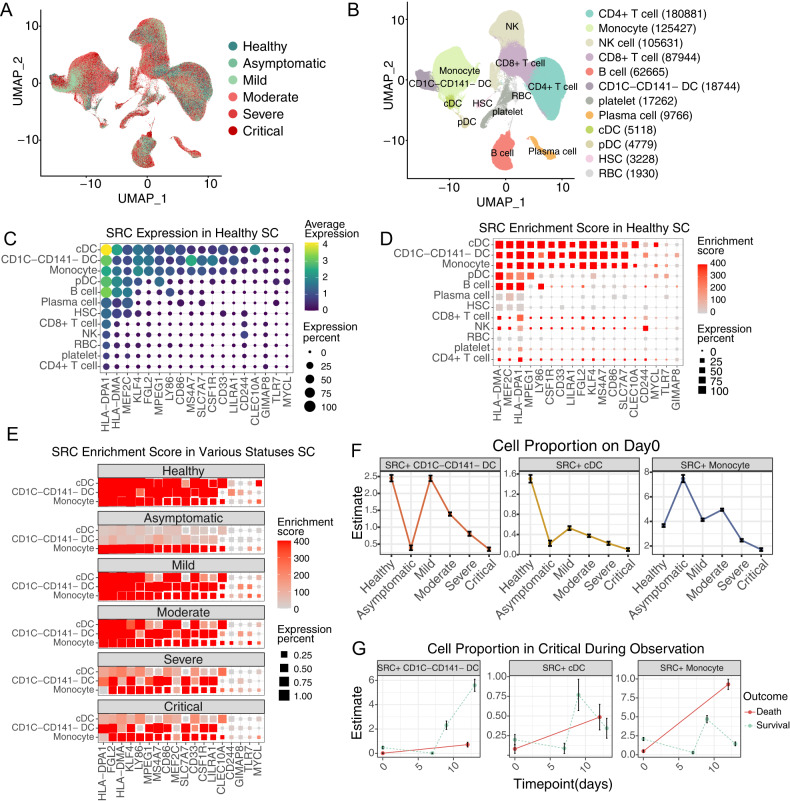
CD1C-CD141- DCs are distinctive and prognostic cell source of SRC genes in COVID-19 scRNA-seq. UMAP dimensionality reduction embedding of healthy and COVID-19 patients peripheral blood mononuclear cells after QC and colored by disease status (**A**) and annotated cell types (**B**). The numbers indicated the cell number of each cell type in (**B**). **C** SRC genes expression dot plot in healthy subjects. The dot color and size indicated the scaled expression level and percentage. **D** SRC enrichment score of each cell type in healthy subjects. The square color and size represented the enrichment score and expression percentage. **E** SRC enrichment score of CD1C-CD141- DCs, monocytes and cDCs across the disease statuses. The square color and size represented the enrichment score and expression percentage. **F** SRC + CD1C-CD141- DCs, monocytes and cDCs proportion of different disease statuses on sample-collection time. The error bar represented the 95% confidence interval and the y axis represented the cell percentage. **G** SRC + CD1C-CD141- DCs, monocytes and cDCs proportion of critical COVID-19 patients during disease progression. The outcome was annotated by line color. The error bar represented the 95% confidence interval. ****P < 0.0001.

To identify the source cells of SRCs, we employed scRNA-seq data of COVID-19 PBMCs. After quality control, a total of 623 375 cells from healthy controls and COVID-19 patients were grouped into 15 clusters by a shared nearest neighbor (SNN) modularity optimization based clustering algorithm (Fig. 3A). The clusters were then annotated into 12 cell types according the cluster gene markers (Fig. 3B, Supplementary Fig. S3A). We then examined the expression level and specificity of SRCs of each cell type in healthy control. 18 of 19 SRCs were detected in COVID-19 scRNA-seq data except MARCHF1. CD1C-CD141-DCs, cDCs and monocytes all exhibited a much higher expression level and percentage compared to other cell populations (Fig. 3C). Fisher’s Exact Test (FET) inspection confirmed that SRCs were monocyte-DC specific (Fig. 3D). Indeed, cDCs, CD1C-CD141-DCs and monocytes had the highest SRC score compared with other cells (Supplementary Fig. S4A). Remarkably, the SRCs were preferred to be expressed by monocytes in asymptomatic COVID-19 patients, while by CD1C-CD141-DCs and monocytes in severe and critical COVID-19 individuals (Fig. 3E). Together, the results indicate that CD1C-CD141-DCs and monocytes were the essential cellular sources mediating heterogeneous responses to COVID-19.

### Enhanced SRCs in Mono-DCs showed T-dependent potential and less severity of COVID-19

To assess SRCs’ function in cells, we arbitrarily divided Mono-DCs into SRC positive (SRC+) or negative (SRC-) groups based on the number of expressed SRCs (median value 8 as the threshold). Interestingly, both the SRC+ Mono-DCs were functionally enriched in antigen processing and presenting, and T cell activation, suggesting a potential T-dependent function, while SRC- Mono-DCs were enriched in electron transport chain (ETC) (Supplemental Figure S4B–D), indicating that the SRCs participated the functional polarization of the monocytes and DCs.

We next focused on the distributions of SRC+ cells in healthy controls and various COVID-19 statuses. We found that the percentages of the SRC+ monocytes and CD1C-CD141-DCs were the majority of SRC+ Mono-DCs in the healthy controls (Fig. 3F). The COVID-19 infection led to gradual reduction of the SRC + CD1C-CD141-DCs and cDCs with the disease severity (from mild to critical) (Fig. 3F). The SRC+ monocytes demonstrated the similar pattern as well (Fig. 3F). Unexpectedly, the asymptomatic COVID-19 individuals had much higher percentage of the SRC+ monocytes compared to all other clinical statuses or Mono-DC subtypes (Fig. 3F). Moreover, the antigen presentation score of asymptomatic SRC+ monocytes were higher than that of SRC+ monocytes from other COVID-19 statuses (Supplementary Fig. S4E). Thus, SRC in monocytes might introduce a specific antigen dependent action and contributed to the COVID-19 asymptomatic phenotype.

It is noticed that the average expression levels of SRCs in the individual SRC+ Mono-DCs showed limited differences across the different clinical statuses (Supplementary Fig. S4F) while the SRC scores in overall Mono-DC populations were gradient decreased with COVID-19 severity (Supplementary Fig. S4G). These findings were consistent with the bulk transcriptome results and supported the notion that it is the reduction of SRC+ Mono-DCs that is vital in COVID-19 progression. To test this hypothesis, we asked whether the recovery of the SRC+ Mono-DC proportion was critical for the survival of critical COVID-19 patients. We found that it was the SRC + CD1C-CD141-DCs, rather than the cDCs or monocytes, which was the key mediators dramatically elevating the survival of critical COVID-19 patients (Fig. 3G).

### Poor IFN response and immune-crosstalk of CD1C-CD141-DCs in critical COVID-19

**Fig. 4:**
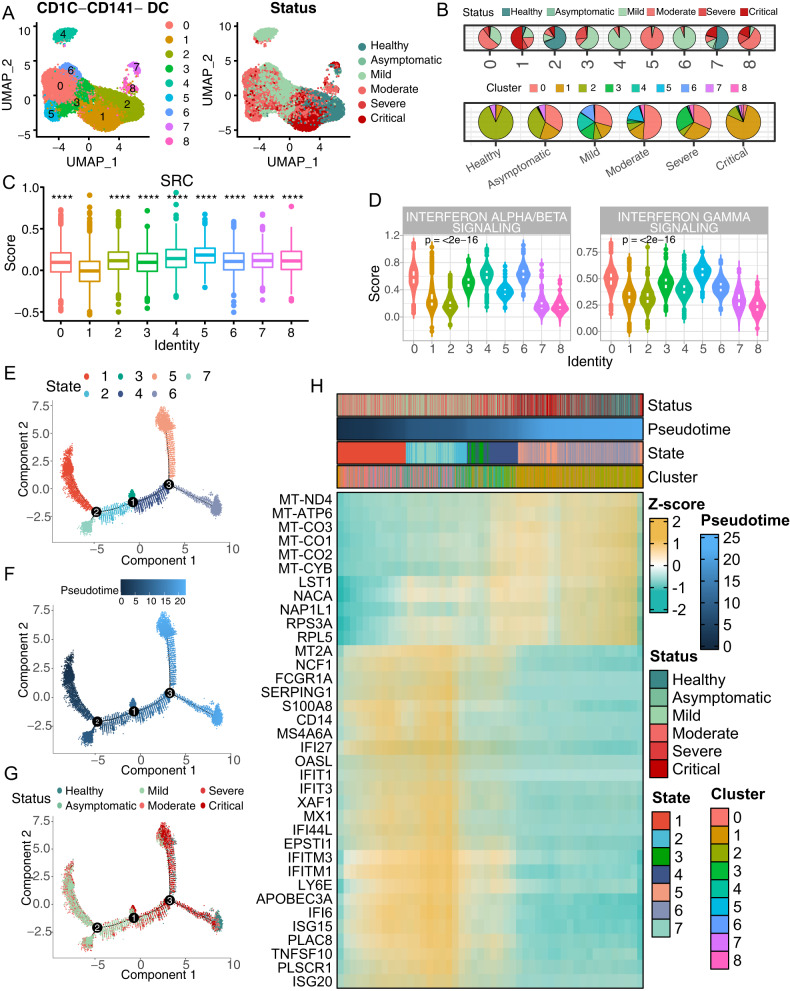
CD1C-CD141- DCs subcluster of critical COVID-19 patients is IFN- unresponsive. **A** UMAP embedding visualization of CD1C-CD141- DC sub-clusters which colored by cluster identity (left) and disease status (right). **B** Pie chart of CD1C-CD141- DCs composition in each cluster (top) and disease status (bottom), respectively. **C** Boxplot of SRC genes score among each CD1C-CD141- DC sub-clusters. Cluster1 was compared to the other clusters. **D** Violin plot of Interferon signaling score across each CD1C-CD141- DC sub-clusters. Kruskal-Wallis test *p* value was annotated on the top left. UMAP visualization of CD1C-CD141- DC trajectories colored by state (**E**), pseudotime (**F**), and disease status (**G**). **H** Representative heatmap of differentially expressed and correlated with pseudotime genes.

To illustrate the functional features of CD1C-CD141-DCs along with COVID-19 severity, we divided CD1C-CD141-DCs into nine subclusters (Fig. 4A). The majority CD1C-CD141-DCs in the healthy controls (86.7%) and the critical COVID-19 (78.1%) were found in cluster2 and 1, respectively, which were adjacent in silicon, indicating an overall similarity in gene expression between the cells that were either uninfected or unresponsive (Fig. 4A, B). The critical-COVID-19-dominant cluster1 had the lowest SRC score among the clusters (Fig. 4C). The status-integrated SRC score decreased with increasing disease severity, and the critical COVID-19 had the lowest score (Supplementary Fig. S5E), indicating a strong association between SRCs expression of CD1C-CD141-DCs and COVID-19 progression.

To deduct the inward cellular signaling in CD1C-CD141-DCs, we first applied the transcription factor (TF) activity prediction. STAT1 and IRF8 were recognized as the most potent TFs according to the normalized enrichment score (NES) (Supplementary Fig. S5A, B). In scRNA-seq data, we confirmed that the cluster1 CD1C-CD141-DCs was inactivated in STAT1 and IRF8 regulons activity (Supplementary Fig. S5C). The predicated target genes of STAT1 and IRF8 in scRNA-seq were highly enriched in type I and II IFN signaling, antigen presenting and T cell activation, which were down-expressed in both cluster1 (critical) and 2 (healthy) of CD1C-CD141-DCs (Supplementary Fig. S5C, D). The scores of IFN signaling pathways confirmed the inactivity of these pathways in critical COVID-19 patients and healthy controls (Supplementary Fig. S5E).

For chasing the trajectory of the subclusters of CD1C-CD141-DCs, we then applied pseudotime analysis. Consistent with the UMAP results (Fig. 4A), there was great similarity between CD1C-CD141-DCs from healthy controls and critical COVID-19 patients in trajectory branches and pseudotime (Fig. 4E–G). The interferon response genes and mitochondrial genes were negative- and positive- correlated with pseudotime, respectively (Fig. 4H), suggesting these genes as the major contributors in CD1C-CD141-DCs differentiation. Since the oxidative phosphorylation suppression is the signature of DCs activation [32, 33], this result further supported that the CD1C-CD141-DCs low-responsive or un-stimulated status in the critical COVID-19 or healthy controls, respectively. Interestingly, the critical-COVID-19-dominant cluster1 was additionally suppressed in TNF signaling (TNFSF10 and TNFSF13B) compared to that of the healthy-control-dominant cluster2 (Supplementary Fig. S4A).

**Fig. 5:**
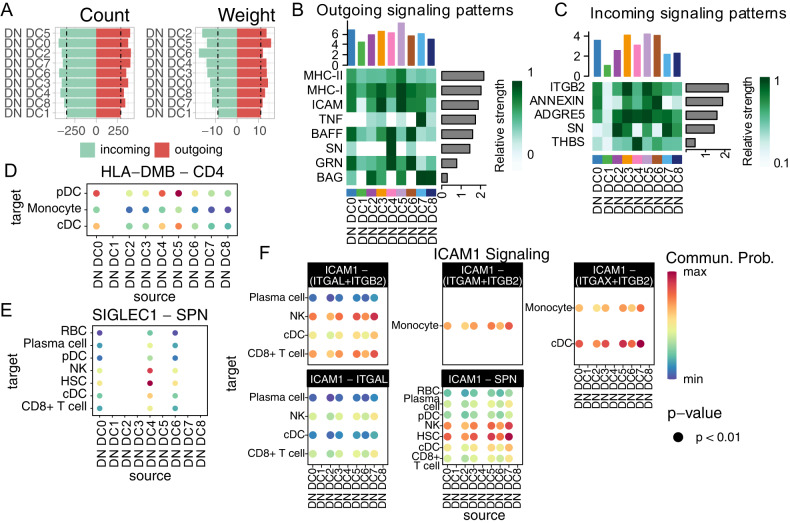
CD1C-CD141- DCs subcluster of critical COVID-19 patients is immune-crosstalk quiet with other immune cells. **A** Barplot of CD1C-CD141- DC subclusters overall signaling pathway count (left) and weight (right). The bar was colored by the incoming and outgoing status. DN DC: CD1C-CD141- DC. Representative heatmap of CD1C-CD141- DC outgoing (**B**) and incoming (**C**) signaling relative strength. Representative dot plot of CD1C-CD141-DC outgoing signaling, including HLA-DMB–CD4 (**D**), SIGLEC1-SPN (**E**) and ICAM1 (**F**) signaling. The dot size indicated the *p* value and the color represent communication probability. DN DC represents CD1C-CD141-DC.

Interestingly, among the nine clusters, the critical COVID-19 dominant cluster 1 showed the lowest count and weight of both outgoing and incoming signaling, while cluster 2 (healthy dominant) was among the highest ranking in both (Fig. 5A), indicating that the CD1C-CD141-DCs in critical COVID-19 were communication defective and were completely different to those of the healthy controls. Specifically, the critical COVID-19 dominant cluster1 showed a decrease in incoming signaling for ITGB2, ANNEXIN, ADGRE5, SN, and THBS, and a decrease in outgoing signaling for MHC-II, MHC-I, ICAM, TNF, BAFF, SN, GRN, and BAG (Fig. 5B, C), thus incompetent in immune-crosstalk. We also found that the interaction of HLA-DMB-CD4 from cluster1 to other Mono-DCs was completely missing (Fig. 5C). CD4 signaling in monocyte was previously suggested to promote their differentiation into macrophages and phagocytosis [34] while CD4+ monocyte were reduced in COVID-19 [35]. In our study, we observed a significant reduction in CD4 expression in monocytes of severe or critical COVID-19 patients as well (Supplementary Fig. S6B).

**Fig. 6:**
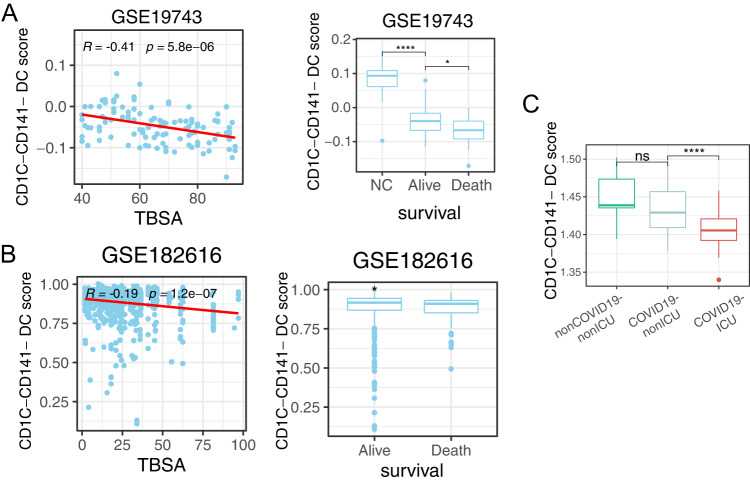
CD1C-CD141- DCs gene markers were related to clinical traits in burn and COVID-19 bulk transcriptomes. CD1C-CD141- DCs gene marker scores correlated with TBSA (left) and prognosis (right) in burns transcriptome datasets of GSE19743 (**A**) and GSE182616 (**B**). **C** Boxplot of CD1C-CD141- DCs gene marker scores in COVID-19 transcriptome dataset. *P* values were adjusted by FDR. *P < 0.05, **P < 0.01, ***P < 0.001.

In addition, the interaction between SIGLEC1 and SPN (CD43), which was crucial in T cell proliferation, differentiation, migration and activation [36, 37], was disrupted in cluster1 with CD8 + T cells (Fig. 6E). Moreover, the ICAM1 signaling, interacted with ITGAL, ITGB2 and SPN, was broadly inactive in cluster1 with CD8 + T cells (Fig. 6F). The interaction of ICAM1 with ITGAL was vital to activate T cells through forming a defined zone in immunological synapse [38, 39]. Indeed, CD8 + T cells rather than CD4 + T cells were decreased in severe and critical COVID-19 compared with moderate status (Supplementary Fig. S3C). The other signaling pathways were observed different in cluster1, involving TNF, ADGRE5-CD55, ANXA1-FPR2 and THBS1-CD36 (Supplementary Fig. S6C–F), which needed further research.

### CD1-CD141-DC score was related to burns and COVID-19 transcriptome datasets

Finally, to assess whether the single cell results were indeed related to the clinic traits in burns and COVID-19 transcriptome datasets, the CD1-CD141-DC marker gene score was calculated in peripheral blood transcriptome datasets of burns and COVID-19. The CD1-CD141- DC score was negatively correlated to TBSA in burn datasets and was suppressed in patients with poor prognosis. (Fig. 6A, B), suggesting that the burns could induce the depletion of CD1-CD141-DCs. Also, the CD1-CD141-DC score shared a similar pattern with the SRCs in the COVID-19 transcriptome dataset (Fig. 6A), specifically reduced in critical COVID-19 patients (Fig. 6C). These findings argued that CD1-CD141-DCs were the critical prognostic cell population of SRCs in response to burns and COVID-19.

## Discussion

We hypothesized that different stressors may elicit a shared molecular response. To test it, we identified a CD86-centered network that exhibited coordinated expression and PPI patterns in the transcriptome of the peripheral blood from burn-injured patients using WGCNA. This network associated with the innate and adaptive immune response, including pattern recognition, antigen presentation and T cell co-stimulation. Remarkably, the network genes were robust in expression behavior and clinical relevance, supporting our initial hypothesis at least in the context of burns and COVID-19.

It is known that the incidence of sepsis in burn patients, typically resulting from infection, is higher (8–42.5%) than trauma (2.4–16.9%) or even critical care patients (19–38%) [40]. Burn injuries can also increase patients’ susceptibility to infection by compromising their metabolic and immunological defenses [7, 41]. T cell exhaustion is always combined with server burn injury while the precise molecular mechanism remains not fully investigated. Our results demonstrated that CD86-centered immune processes were disrupted across the burn initiation, progression and outcome. The genes, such as CD86, CD1C and HLA-DPA1 etc., which were essential to T cell proliferation and activation, were in high degree of SRC network.

The analysis of COVID-19 scRNA-seq demonstrated that the Mono-DCs in the circulation were the cellular host of SRC genes. The CD1C-CD141-DCs were molecularly characterized of enhanced IFN-I and anti-virus compared to other DC populations [42]. Interestingly, CD1C-CD141-DCs were reported to be the most sensitive to exercise-induced mobilization which increased up to 167% after exercise [43], indicating that CD1C-CD141-DCs were potentially the fast responders and effectors during stress. Our study also found that CD1C-CD141-DCs deficiency was linked to progression and poor prognosis of COVID-19. Unexpectedly, in the asymptomatic patients, it was the monocytes that were significantly increased with enhanced SRCs expression, while the numbers of CD1C-CD141-DCs and cDCs were greatly reduced. However, the casual inference of CD1C-CD141-DCs with COVID-19 prognosis, as well as monocytes in asymptomatic patients of COVID-19, and the specific mechanism of mobilization relative cell types needed further investigations.

Besides the quantity restriction, the function of CD1-CD141-DCs in critical COVID-19 patients was “poisoned”, which was captured as poor IFN response, high mitochondrial metabolism and immune cross talk quiescent. The impaired IFN signaling, including delayed responses and genetic mutations, was essential for virus replication and tissue damages in COVID-19 [44–46]. Moreover, the sensitivity and interferon-stimulated genes (ISGs) expression prolife of IFN varied among different cell types [44]. DCs stimulated by IFNs orchestrate the innate and adaptive immune responses which transform to the phenotype of high antigen presentation and co-stimulation [47, 48]. Our studies confirmed this molecular responding pattern and further recognized CD1C-CD141-DCs as the vital subtype of the Mono-DC population in COVID-19 progression and prognosis. Mitochondrial metabolism, including ETC and oxidative phosphorylation, was suppressed in monocytes/DCs during their activation [33]. CD1C-CD141-DCs in critical COVID-19 and healthy controls both highly expressed ETC components, indicating their unresponsiveness and un-stimulatory status, respectively. Given the fact that the CD1C-CD141-DCs from critical COVID-19 was largely limited to cluster1 which were characterized as immune-crosstalk quiescent, we suggested that the dysfunction of CD1C-CD141-DCs played a key role in critical COVID-19 development and prognosis, though the multi-cell communication patterns in various clusters were complicated likely involving combinatory mechanisms functionally. More studies are guaranteed. Also, in burns condition, the CD1-CD141-DC score shared a similar pattern with the SRCs in the COVID-19, suggesting these DCs playing a key role in burns prognosis as well, which requires further studies.

## Materials and methods

### Data collection and preprocessing

The peripheral blood transcriptome expression profiles and clinical traits of burns patients, including GSE19743 and GSE182616, were downloaded from GEO database (https://www.ncbi.nlm.nih.gov/geo) by R package GEOquery (Version 2.62.2) [49]. The transcriptome expression data was quantile-normalized for further analysis. The peripheral blood leukocytes RNA-sequencing of patients with or without COVID-19 and related clinical information (GSE157103) were downloaded from GEO database. The scRNA-seq data of COVID-19 patients and healthy control were downloaded from CELLxGENE website (https://cellxgene.cziscience.com/collections/ddfad306-714d-4cc0-9985-d9072820c530).

### Bioinformatics analyses

The detailed bioinformatics analyses were available in Supplementary Method.

### Statistical analyses

R (Version 4.1.2) was applied to all statistical tests. Pearson and Spearman correlation analyses were done by R. The statistical analysis of single sample gene enrichment analysis (ssGSEA) score in bulk transcriptome between different disease status were achieved by Wilcoxon signed-rank test. *P* values of multiple comparisons were adjusted by “FDR”. The statistical analysis of single-cell score among the different cell types or clusters was determined by Kruskal-Wallis test. All *P* values were considered significant if <0.05.

## Supplementary information

Supplemental Figures_legends

Supplementary Method

Supplemental_Fig_S1

Supplemental_Fig_S2

Supplemental_Fig_S3

Supplemental_Fig_S4

Supplemental_Fig_S5

Supplemental_Fig_S6

## Data Availability

The transcriptome datasets of burns and COVID-19 can be found in GEO database (https://www.ncbi.nlm.nih.gov/geo/) through the accession numbers of GSE19743, GSE182616 and GSE157103. The scRNA-seq data of COVID-19 patients and healthy control was downloaded from CELLxGENE website (https://cellxgene.cziscience.com/collections/ddfad306-714d-4cc0-9985-d9072820c530).

## Code availability

All codes used in this study are available upon request.
