# Supplementary material for: A burns and COVID-19 shared stress responding gene network deciphers CD1C-CD141- DCs as the key cellular components in septic prognosis
Source: Cell Death Discov. 2023 Jul 24;9:258. doi: 10.1038/s41420-023-01518-7 (PMC10366195; doi:10.1038/s41420-023-01518-7)
Supplement: Supplementary file 1 — Supplemental Figures_legends [file 41420_2023_1518_MOESM1_ESM.docx]

**Supplemental figure legends**

**Supplemental Figure S1. Workflow of investigation.** SRC, stress-response core.

**Supplemental Figure S2. Identification and validation of robust gene co-expression module among burns datasets.**

(A) Spearman correlation heatmap of module eigengenes with clinical traits in burns dataset GSE19743.

(B) Pearson correlation heatmap of skyblue module genes of GSE19743 and GSE182616 datasets. The left-bottom and right-top part of heatmap is based on GSE19743 and GSE182616, respectively.

(C) Barplot of DGCA based on skyblue module genes between GSE19743 and GSE182616. Each gene pair is classed as positive (+), negative (-) and non-significant (0) and grouped by combination of GSE19743 and GSE182616 datasets.

(D) Barplot of DGCA based on skyblue PPI-hub genes between GSE19743 and GSE182616. Each gene pair is classed as positive (+), negative (-) and non-significant (0) and grouped by combination of GSE19743 and GSE182616 datasets. * P < 0.05, ** P < 0.01, *** P < 0.001.

**Supplemental Figure S3. COVID-19 single-cell RNA sequencing prolife.**

(A) Dotplot of cell marker genes in COVID-19 scRNA-seq. The dot color and size represents average expression and expressed percentage of each cell type.

(B) Cell proportion of each cell type among different disease statuses on sample-collection time. The error bar represented the 95% confidence interval and the y axis represented the cell percentage.

**Supplemental Figure S4. Mono-DC wing was the major cell source of SRC genes.**

(A) Boxplot of SRC genes score among each cell types in healthy subjects.

(B-D) DEGs GO enrichment of SRC+/- CD1C-CD141- DCs (B), cDCs (C) and monocytes (D).

(E) MHC-II Antigen presentation score of representative SRC+ Mono-DCs. SRC+ monocytes in asymptomatic subjects were compared to the other sub SRC+ cells. Wilcoxon test was applied and p value was adjusted by FDR.

(F) SRC genes expression dot plot in SRC+ Mono-DCs across disease statuses. The dot color and size indicated the scaled expression level and percentage.

(G) Boxplot of SRC genes score across disease statuses in CD1C-CD141- DCs, monocytes and cDCs. * P < 0.05, ** P < 0.01, *** P < 0.001, **** P < 0.0001.

**Supplemental Figure S5. CD1C-CD141- DCs subcluster of critical COVID-19 patients is IFN pathway inactivated.**

(A) Barplot of top 24 TF regulons of SRC genes. The color indicated the max NES of TF.

(B) Network of top 24 TF and their target genes. The node color was annotated by TF and target genes. The node size represented the degree of each node.

(C) Heatmap of predicted STAT1 and IRF8 regulon activity and targeted gene expression of subclusters of CD1C-CD141- DCs. The antigen-presentation- and IFN- related genes were marked on the left side.

(D) Representative GO enrichment barplot of STAT1 and IRF8 targeted genes.

(E) Boxplot of SRC (left), IFN alpha/beta (middle) and gamma (right) signaling score of CD1C-CD141- DCs across the different statuses. The critical COVID-19 was employed as the referent group. * P < 0.05, ** P < 0.01, *** P < 0.001, **** P < 0.0001.

**Supplemental Figure S6. CD1C-CD141- DCs subcluster of critical COVID-19 patients is immune quiescent with other cells.**

(A) Dotplot of representative DEGs of IFN- and TNF- pathway related genes across subclusters of CD1C-CD141- DCs. The dot color and size indicated the average expression level and expressed percentage of the relative cluster.

(B) Boxplot of CD4 expression across disease statuses in SC-RNA sequencing. Critical subjects were compared to the other subjects. P values were adjusted by FDR.

(C-F) Representative dot plot of DN DC incoming signaling, including TNF (C), ADGRE5-CD55 (D), ANXA1-FPR2 (E) and THBS1-CD36 (F). The dot size indicated the p value and the color represent communication probability. DN DC represents different subclusters of CD1C-CD141-DCs. DN DC represents CD1C-CD141-DC. * P < 0.05, ** P < 0.01, *** P < 0.001, **** P < 0.0001.
