# Supplementary material for: A burns and COVID-19 shared stress responding gene network deciphers CD1C-CD141- DCs as the key cellular components in septic prognosis
Source: Cell Death Discov. 2023 Jul 24;9:258. doi: 10.1038/s41420-023-01518-7 (PMC10366195; doi:10.1038/s41420-023-01518-7)
Supplement: Supplementary file 2 — Supplementary Method [file 41420_2023_1518_MOESM2_ESM.docx]

**Supplementary Methods**

**1. WGCNA**

WGCNA (Version 1.71) was performed to identify crucial gene co-expression modules related to clinical traits on GSE19743 [1]. First, the function “goodSamplesGenes” in R package WGCNA was used to confirm the input genes and samples were suitable, and none outliers were identified. Secondly, Pearson’s correlation analysis of all pairs of genes was used to construct an adjacency matrix. After that, the adjacency matrix was used to build a scale-free network based on a soft-thresholding parameter β which is 8 in the current case, which enhanced strong correlations between genes and penalized weak correlations. Then the matrix was turned into a topological overlap matrix (TOM) to measure the network connectivity of a gene, which was defined as the sum of its adjacency with all other genes. To classify genes with similar expression patterns into gene modules, the average linkage hierarchical clustering was conducted according to the TOM-based dissimilarity measure with a minimum size (gene group) of 30 for the genes dendrogram and 0.1 for mergeCutHeight. The non-co-expressed genes were included in the “gray” module. The relationships of co-expressed gene modules with clinical traits, such as TBSA, survival status and time point, were analyzed.

**2. Differential Gene Correlation Analysis (DGCA)**

R package DGCA (Version 1.0.2) was employed to detect module robustness by comparison the differences in the correlations of gene pairs between distinct biological conditions. Briefly, DGCA calculates the gene pair correlation in each condition and normalizes correlation coefficients by Fisher z-transformation [2]. The finial gene pair correlation status in each condition is determined by empirical p-values computed via permutation testing. Each gene pair is classed as positive (+), negative (-) and non-significant (0) and grouped by combination of specific condition. The function “ddcorAll” was applied in this section.

**3. Single Sample Gene Set Enrichment Analysis (ssGSEA)**

The ssGSEA was carried by R package GSVA (Version 1.42.0) with the function gsva in which the parameter method was set to ‘ssgsea’ [3].

**4. DEGs and enrichment analysis**

R package limma (Version 3.50.3) was applied to identify DEGs with the threshold of |logFC| >1 and p-value <0.05 [4]. The module gene enrichment analysis was carried out by the R package clusterProfiler (Version 4.2.2) [5]. Gene Ontology (GO), including biological process (BP), cellular components (CC) and molecular function (MF), and Kyoto Encyclopedia of Genes and Genomes (KEGG) were analyzed. The p-value <0.05 was considered as a significant outcome.

**5. Transcription factors analysis in bulk transcriptome**

R package RcisTarget (Version 1.14.0) was employed to predict transcription factors of skybklue core genes based on hg19-tss-centered-10kb-7species.mc9nr.feather gene-motif rankings database [6]. Motifs were annotated to TFs based on the pathway enrichment analysis, and the highest normalized enrichment score (NES) was picked up for each TFs. The transcription regulation network was visualized by gephi software (Version 0.9.7).

**6. Single-cell analysis**

**Single-cell sequencing data download, preprocessing and cell annotation.** The COVID-19 single-cell sequencing data was obtained from CELLxGENE website. The cells from COVID-19 patients and healthy control were selected for further analysis. Cells with fewer 200 genes expression and >10% mitochondrial reads were removed in quality control, Then, the SC-RNAseq data was normalized, scaled and dimension-reduction (PCA and UMAP). We set dim=15 to in “FindNeighbors” function to construct nearest-neighbor graph according to the standard deviations of the principle components which was visualized by “ElbowPlot” function. The cells were divided into 15 populations by “FindClusters” function with resolution=0.2. RunUMAP was applied for visualization. The cell type was determined by the cluster marker majorly referred to CellMarker database (<http://xteam.xbio.top/CellMarker/>) as follows, CD4+ T cell (CD3D, CD3E, CD14, LTB, IL7R, MAL), CD8+ T cell (CD3D, CD8A, CD8B, GZMK, NKG7), NK (GNLY, GZMB, NKG7, KLRD1, KLRF1, CD247), B cell (CD79A, CD74, MS4A1, CD19, HLA-DRA, HLA-DQA1), plasma cell (JCHAIN, IGHG1, IGHA1, MZB1, TXNDC5), monocyte (CD14, CD68, S100A12, SERPINA1), pDC (PLD4, ITM2C, LILRA4, IRF7, IRF8, SERPINF1), cDC (CD1C, CD86, HLA-DRA, HLA-DRB5, HLA-DRB1), CD1C-CD141- DC (MS4A7, CFD, LRRC25, FCGR3A, CD68), HSC (CD34, SPINK2, GATA2, MYB, ALDH1A1), RBC (HBB, HBA2, HBA1, HBM), platelet (CD151, GP9, ITGA2B). R package Seurat (Version 4.1.1) was applied in this section [7].

**SRC genes cell location.** The cell-location of SRC genes was determined by expression level and Fisher’s Exact Test (FET) ^[8]^. For FET part, we first identified a list of cells expressing the gene with a threshold count_i > 0 for a given gene i in SRC, then tested how these cells were enriched for the cells in each inferred cell type under the comparison of one-to-rest by FET. Adjusted p value by FDR < 0.05 was considered significance to designate a cell type for the gene. The enrichment score was calculated by –log10(p-adjust). And of nation, if the adjusted p value of some cell types which equaled to 0, then the enrichment score of these cell types was set to 1 while others was 0. R package stats (Version 4.2.1) function “fisher.test” was utilized in this part.

**Single-cell score calculation**. The single-cell score was calculated by Seurat function “AddModuleScore” of gene lists [7]. The search parameter was set to TRUE to match the gene symbols. The gene lists of IFN signaling (IFN alpha/beta and IFN gamma) were downloaded from MSigDB database (<http://www.gsea-msigdb.org/gsea/login.jsp>) with the accession of RECATOME INTERFERON ALPHA/BETA and RECATOME INTERFERON GAMMA.

**Fractional abundance of cell population analysis.** The confidence intervals of relative proportion of different cell types was calculated by R package REdaS (Version 0.9.4) function “freqCI”.

**CD1C-CD141- DC sub cluster analysis.** This section was achieved by R package Seurat [7]. First, CD1C-CD141- DCs were filtered and followed by Seurat pipeline analysis, including normalization, scale and dimension-reduction. The sub clusters of CD1C-CD141- DC were decided by function “FindClusters” which the resolution was set to 0.3.

**Single-cell regulatory network inference and clustering (SCENIC) analysis.** The SCENIC analysis was applied by pySCENIC (version 0.11.2) [6]. The expression matrix of raw UMI counts of CD1C-CD141- DCs was employed in this section. After default data filtering, grnboost2 method was utilized to generate gene regulatory networks. Then, the enriched motifs were determined by “ctx” function in pySCENIC based on cisTarget Human motif database v9 of regulatory features 10 kb centered on the TSS. Finally, enrichment of regulons across single cells was scored by the “aucell” function. The results were visualized by R package ComplexHeatmap (Version 2.10.0) [9].

**Pseudotime analysis.** The CD1C-CD141- DC sub clusters were employed to perform trajectory analysis by R package monocle (Version 2.22.0) [10]. The different genes (q value <0.01) among the COVID-19 status were picked up to order cells which were calculated by function “differentialGeneTest” of monocle. The genes correlated with pseudotime under the threshold of Spearman correlation index > 0.4 or < -0.4 were selected for visualization.

**Cell-cell interaction analysis.** The cell-cell interaction of CD1C-CD141- DC sub cluster and other cells were achieved by R package CellChat (Version 1.4.0) based on public repository of ligands, receptors, cofactors, and their interactions [11]. Briefly, significant interactions of ligand-receptor pairs between cell types were identified by perturbation testing and further constructed communication network. The different incoming and outgoing signals of among CD1C-CD141- DC clusters were systematically analyzed and the specific contributed signaling pathway visualized through bubble plot.

**REFERENCES**

1. Langfelder P, Horvath S. Wgcna: an r package for weighted correlation network analysis. *Bmc Bioinformatics* **9**, 559 (2008). https://doi.org/10.1186/1471-2105-9-559

2. Mckenzie AT, Katsyv I, Song WM, Wang M, Zhang B. Dgca: a comprehensive r package for differential gene correlation analysis. *BMC Syst Biol* **10**, 106 (2016). https://doi.org/10.1186/s12918-016-0349-1

3. Hänzelmann S, Castelo R, Guinney J. Gsva: gene set variation analysis for microarray and rna-seq data. *Bmc Bioinformatics* **14**, 7 (2013). https://doi.org/10.1186/1471-2105-14-7

4. Ritchie ME, Phipson B, Wu D, Hu Y, Law CW, Shi W, Smyth GK. Limma powers differential expression analyses for rna-sequencing and microarray studies. *Nucleic Acids Res* **43**, e47 (2015). https://doi.org/10.1093/nar/gkv007

5. Yu G, Wang L, Han Y, He Q. Clusterprofiler: an r package for comparing biological themes among gene clusters. *Omics* **16**, 284-7 (2012). https://doi.org/10.1089/omi.2011.0118

6. Aibar S, Gonzalez-Blas CB, Moerman T, Huynh-Thu VA, Imrichova H, Hulselmans G, Rambow F, Marine JC, Geurts P, Aerts J, van den Oord J, Atak ZK, Wouters J, Aerts S. Scenic: single-cell regulatory network inference and clustering. *Nat Methods* **14**, 1083-6 (2017). https://doi.org/10.1038/nmeth.4463

7. Hao Y, Hao S, Andersen-Nissen E, Mauck WR, Zheng S, Butler A, Lee MJ, Wilk AJ, Darby C, Zager M, Hoffman P, Stoeckius M, Papalexi E, Mimitou EP, Jain J, Srivastava A, Stuart T, Fleming LM, Yeung B, Rogers AJ, Mcelrath JM, Blish CA, Gottardo R, Smibert P, Satija R. Integrated analysis of multimodal single-cell data. *Cell* **184**, 3573-87 (2021). https://doi.org/10.1016/j.cell.2021.04.048

8. Song WM, Agrawal P, Von Itter R, Fontanals-Cirera B, Wang M, Zhou X, Mahal LK, Hernando E, Zhang B. Network models of primary melanoma microenvironments identify key melanoma regulators underlying prognosis. *Nat Commun* **12**, 1214 (2021). https://doi.org/10.1038/s41467-021-21457-0

9. Gu Z, Eils R, Schlesner M. Complex heatmaps reveal patterns and correlations in multidimensional genomic data. *Bioinformatics* **32**, 2847-9 (2016). https://doi.org/10.1093/bioinformatics/btw313

10. Trapnell C, Cacchiarelli D, Grimsby J, Pokharel P, Li S, Morse M, Lennon NJ, Livak KJ, Mikkelsen TS, Rinn JL. The dynamics and regulators of cell fate decisions are revealed by pseudotemporal ordering of single cells. *Nat Biotechnol* **32**, 381-6 (2014). https://doi.org/10.1038/nbt.2859

11. Jin S, Guerrero-Juarez CF, Zhang L, Chang I, Ramos R, Kuan CH, Myung P, Plikus MV, Nie Q. Inference and analysis of cell-cell communication using cellchat. *Nat Commun* **12**, 1088 (2021). https://doi.org/10.1038/s41467-021-21246-9
