# Supplementary figures and images for: A burns and COVID-19 shared stress responding gene network deciphers CD1C-CD141- DCs as the key cellular components in septic prognosis
Source: Cell Death Discov. 2023 Jul 24;9:258. doi: 10.1038/s41420-023-01518-7 (PMC10366195; doi:10.1038/s41420-023-01518-7)

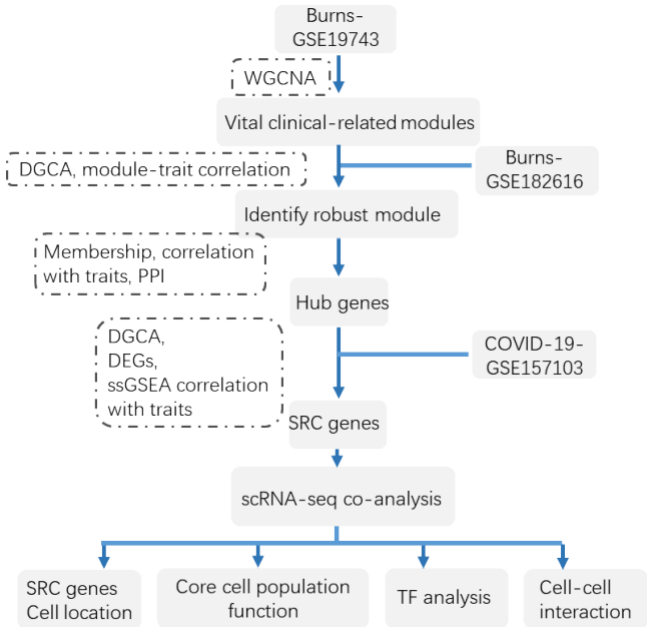

Supplement: Supplementary file 3 — Supplemental_Fig_S1 [file 41420_2023_1518_MOESM3_ESM.pdf]

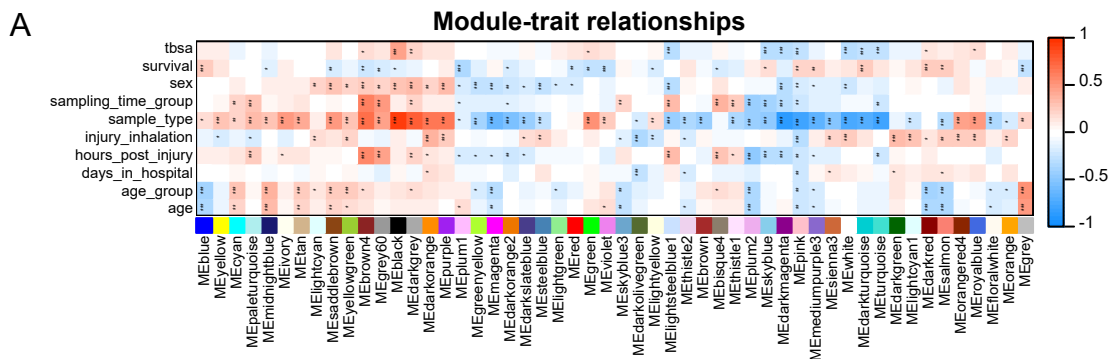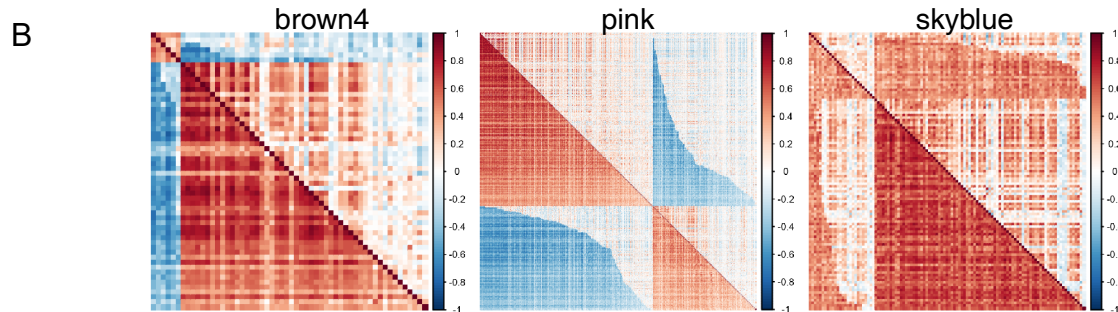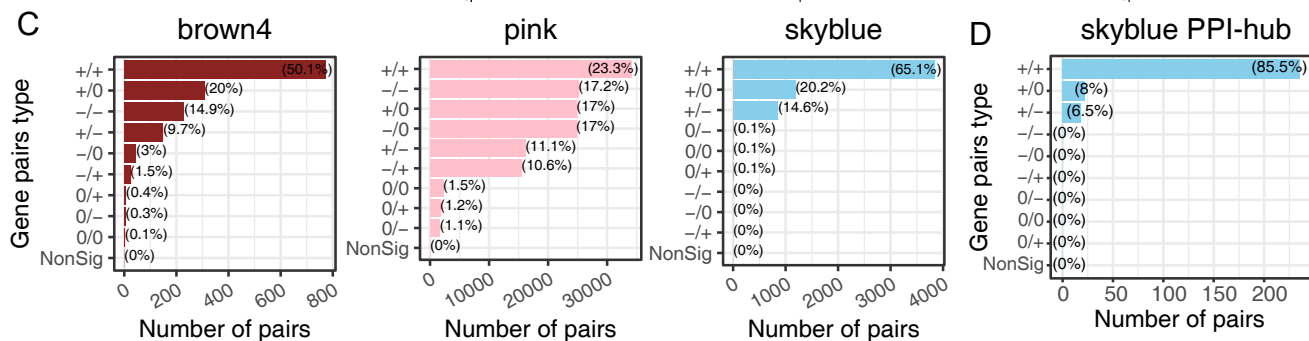

Supplement: Supplementary file 4 — Supplemental_Fig_S2 [file 41420_2023_1518_MOESM4_ESM.pdf]

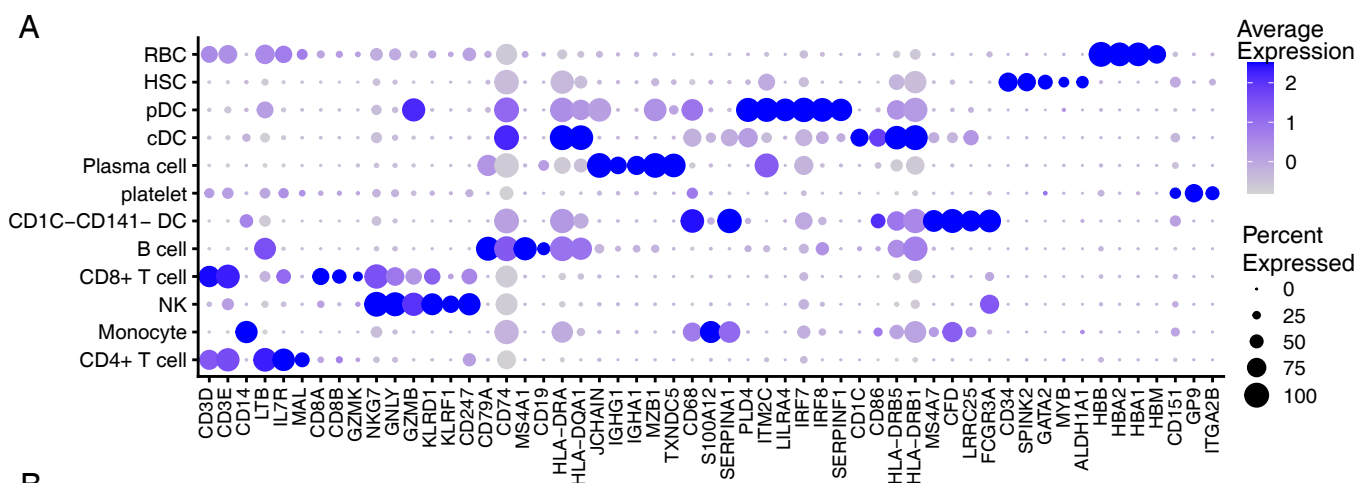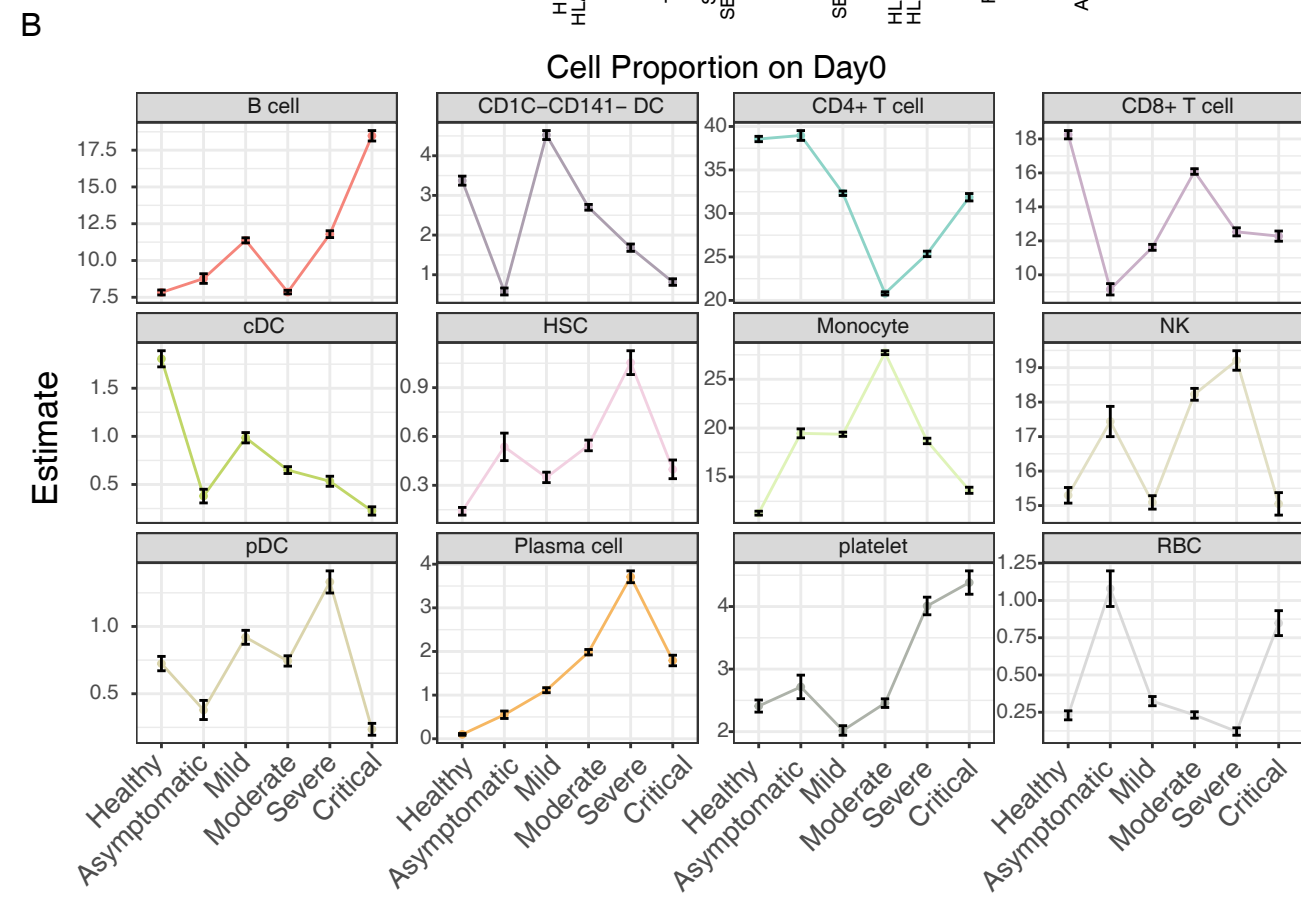

Supplement: Supplementary file 5 — Supplemental_Fig_S3 [file 41420_2023_1518_MOESM5_ESM.pdf]

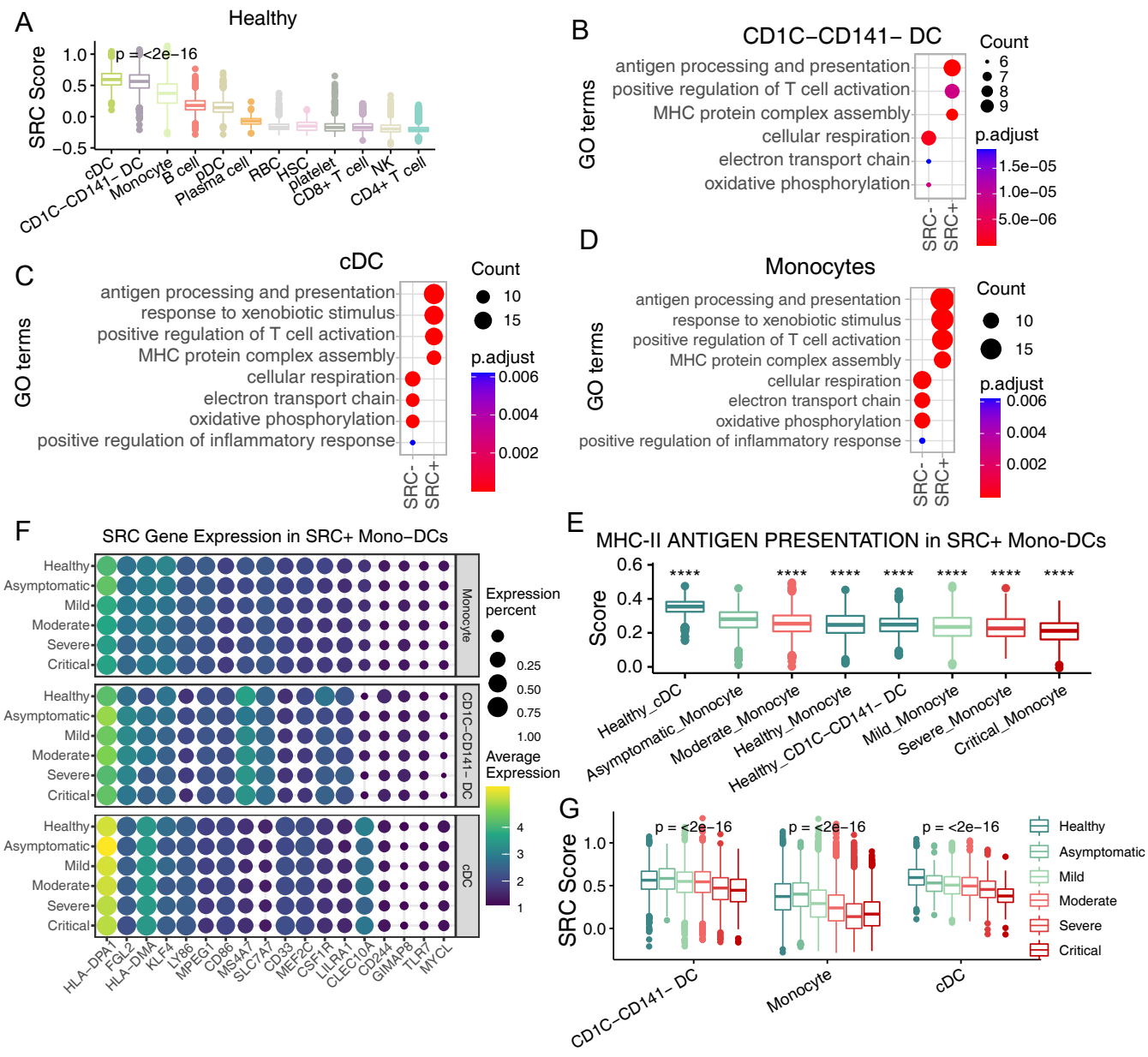

Supplement: Supplementary file 6 — Supplemental_Fig_S4 [file 41420_2023_1518_MOESM6_ESM.pdf]

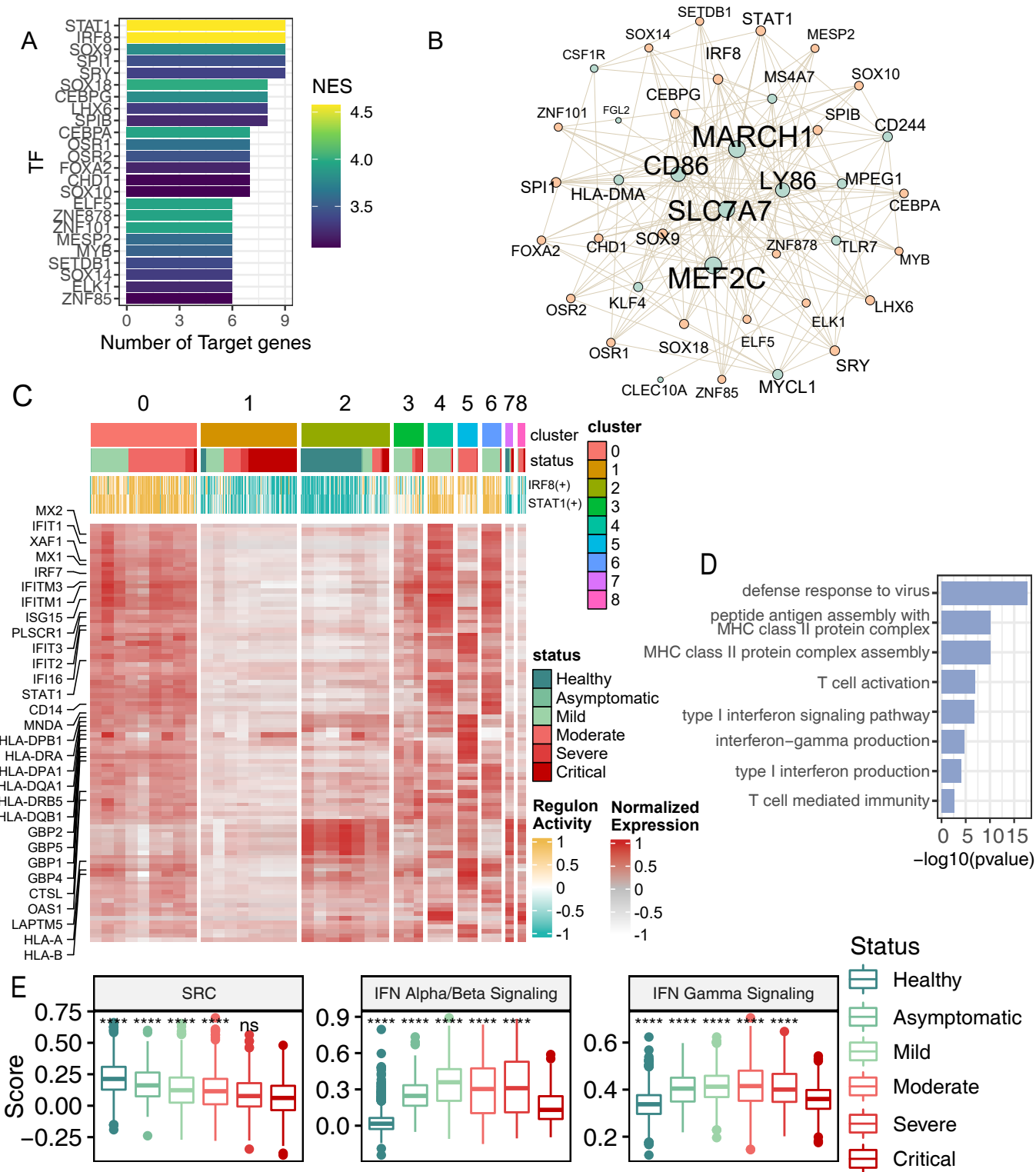

Supplement: Supplementary file 7 — Supplemental_Fig_S5 [file 41420_2023_1518_MOESM7_ESM.pdf]

Identity

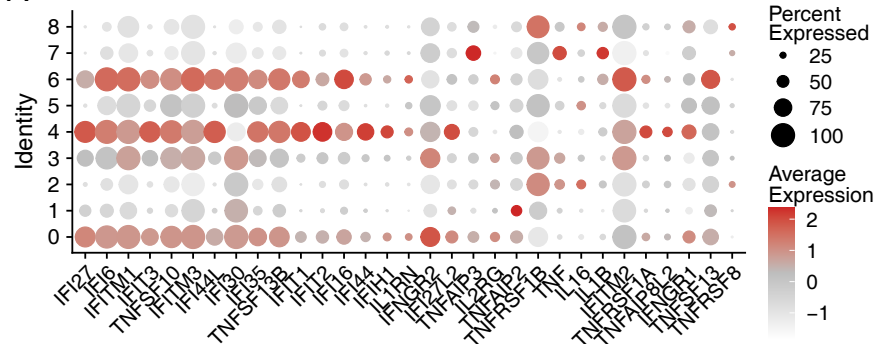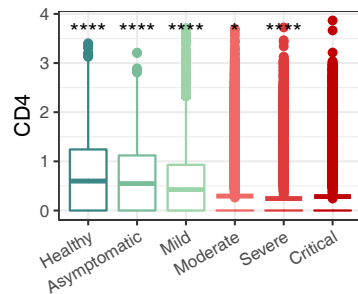

C

## TNF Signaling

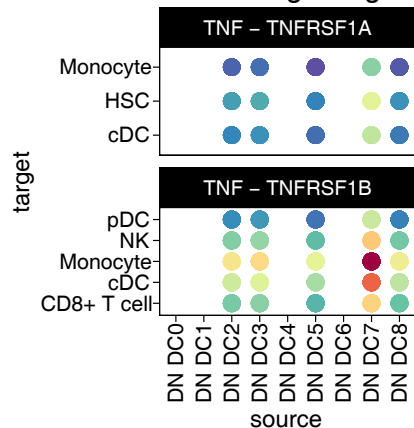

D

ADGRE5 – CD55

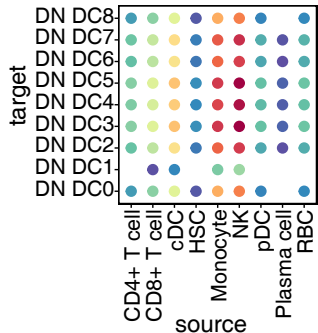

E

ANXA1 – FPR2

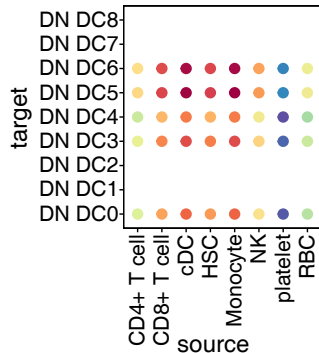

F

## THBS1 – CD36

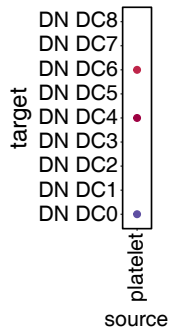

Supplement: Supplementary file 8 — Supplemental_Fig_S6 [file 41420_2023_1518_MOESM8_ESM.pdf]
